# Respiratory extracellular vesicle isolation optimization through proteomic profiling of equine samples and identification of candidates for cell-of-origin studies

**DOI:** 10.1371/journal.pone.0315743

**Published:** 2025-01-24

**Authors:** Elise Hickman, Victoria Carberry, Celeste Carberry, Bethanie Cooper, Angie L. Mordant, Allie Mills, Marina Sokolsky, Laura E. Herring, Neil E. Alexis, Meghan E. Rebuli, Ilona Jaspers, Katie Sheats, Julia E. Rager

**Affiliations:** 1 Curriculum in Toxicology & Environmental Medicine, UNC Chapel Hill, Chapel Hill, North Carolina, United States of America; 2 Department of Environmental Sciences and Engineering, Gillings School of Global Public Health, UNC Chapel Hill, Chapel Hill, North Carolina, United States of America; 3 Department of Clinical Sciences, North Carolina State University College of Veterinary Medicine, Raleigh, North Carolina, United States of America; 4 UNC Michael Hooker Proteomics Core, UNC Chapel Hill, Chapel Hill, North Carolina, United States of America; 5 Center for Nanotechnology in Drug Delivery, UNC School of Medicine, Chapel Hill, North Carolina, United States of America; 6 Department of Pharmacology, UNC Chapel Hill, Chapel Hill, North Carolina, United States of America; 7 Department of Pediatrics, UNC School of Medicine, Chapel Hill, North Carolina, United States of America; Texas Tech University Health Science, Lubbock, UNITED STATES OF AMERICA

## Abstract

Growing evidence supports the importance of extracellular vesicle (EV) as mediators of communication in pathological processes, including those underlying respiratory disease. However, establishing methods for isolating and characterizing EVs remains challenging, particularly for respiratory samples. This study set out to address this challenge by comparing different EV isolation methods and evaluating their impacts on EV yield, markers of purity, and proteomic signatures, utilizing equine/horse bronchoalveolar lavage samples. Horses can serve as effective translational animal models for respiratory studies due to similarities with human immune responses, shared environmental exposures, and naturally occurring respiratory diseases including asthma. Further, horses are long-lived large animals that allow for longitudinal sample collection, and provide large sample volume and cell yield, which are particularly useful since EV research is commonly limited by low sample yields. Here, EVs were isolated from horse bronchoalveolar lavage fluid (BALF) using four different methods (ultracentrifugation, microcentrifugation, and two sizes of size exclusion chromatography columns) and characterized by measuring particle counts, EV purity, total protein yield, and proteomic cargo, with a specific focus on vesicle surface marker expression potentially informing cell type of origin. We found that size exclusion chromatography yielded the highest particle counts, greatest EV purity markers and elevated vesicle surface marker expression. Overall proteomic profiles differed across isolation methods, with size exclusion chromatography clustering separately from centrifugation. Taken together, our results demonstrate that different isolation methods impact characteristics of EVs, notably that size exclusion chromatography, compared to centrifugation methods, resulted in higher EV purity and better characterized proteomic diversity, including information on EV cell-of-origin. This is the first study to characterize proteomic profiles of EVs following different isolation methods using equine BALF. The results of this study will pave the way for future studies using equine and human samples to characterize respiratory tract EVs.

## Introduction

Extracellular vesicles (EVs) are membrane-bound small particles released by cells constitutively and in response to perturbation, transferring molecular signals between cells and across tissues [1–3]. Numerous studies implicate EVs as critical mediators of pathological processes such as cancer, metabolic disorders, and lung inflammation and diseases [3, 4]. In the respiratory tract, changes in EV concentration and molecular cargo have been associated with acute respiratory distress syndrome, asthma, chronic obstructive pulmonary disease, pulmonary fibrosis, and respiratory toxicant exposures [3, 5–10]. For example, EVs have been demonstrated to play a role in signaling related to airway remodeling [11], and EV release is increased following exposure to respiratory toxicants such as PM_2.5_, cigarette smoke, and asbestos in multiple systems and organisms [12].

Numerous methods including ultracentrifugation, size exclusion chromatography (SEC), density gradients, fluid flow-based separations, charge-based separations, and commercialized kits have been utilized to isolate EVs from biological samples [13, 14]. Each of these methods offers distinct advantages and disadvantages in terms of yield, specificity, and applicability to different biological matrices and EV subtypes [13]. Furthermore, within this field there is an ongoing effort to optimize and validate protocols that result in replicable EV isolation across biological samples and experimental conditions. The Minimal Information for Studies of Extracellular Vesicles (MISEV) guidelines have been instrumental in recommending best practices and advocating for transparency to increase reproducibility in EV research [15]. However, establishment of methods for isolating and characterizing EVs remains challenging due to limitations related to 1) determining the specific cell source(s) of EVs, 2) acquiring adequate EV yield, particularly for respiratory tract samples, 3) effectively measuring EV cargo, particularly for molecules requiring higher particle yield, such as proteins (in contrast with microRNAs [miRNAs], which can be amplified from smaller amounts of source material), and 4) improving reproducibility given that details are often limited across publications. To date, the majority of respiratory tract EV studies have focused on miRNAs as cargo [16, 17]. Characterizing additional EV cargo such as proteins, lipids, and metabolites represents a critical knowledge gap in the field of respiratory tract EVs.

One of the biggest challenges in EV research, particularly for respiratory samples, is acquiring sufficient sample volume with which to perform downstream analyses. For example, studies of human bronchoalveolar lavage fluid (BALF) often use 20–100 mL of unprocessed fluid [18–20], which is a much larger volume than typically aliquoted in biorepositories, or do not clearly describe the volume of sample used for EV isolation [21, 22], limiting feasibility and reproducibility of BALF EV studies. Studies of mouse respiratory tract EVs require animal sacrifice and oftentimes pooling of samples, in addition to limitations in interpretability given the differing physiology of mouse and human respiratory tracts [23, 24]. Horses represent a unique and feasible model organism to overcome these limitations because 1) horses develop respiratory diseases found in humans (asthma) with similar pathophysiology [25, 26]; 2) the high volume of horse BALF, and subsequent EV yield allows for robust experimental methods optimization in contrast with small, lower-yield aliquots of human or mouse respiratory samples that are more invasive to collect; 3) horses are exposed to ambient air pollution as they graze in their outdoor pastures, allowing for the investigation of real-world exposure scenarios; and 4) horse BALF collection is minimally invasive and therefore can be collected longitudinally.

In addition to quantifying EV yield, the characterization of proteomic cargo and cell surface markers from respiratory tract EVs represents a critical research gap towards understanding of both basic biological processes and responses to diseases and toxicants mediated by EVs in the respiratory tract. In this study, we leveraged horse BALF to optimize methods for respiratory tract EV isolation and to characterize respiratory EV proteomic profiles—including markers of EV purity, contamination, and cell surface markers—across four different EV isolation methods. These findings will improve our understanding of the communication capacity of EVs and their cells of origin, paving the way for future studies investigating EV-specific mechanisms of respiratory responses and disease outcomes.

## Materials and methods

**Fig 1:**
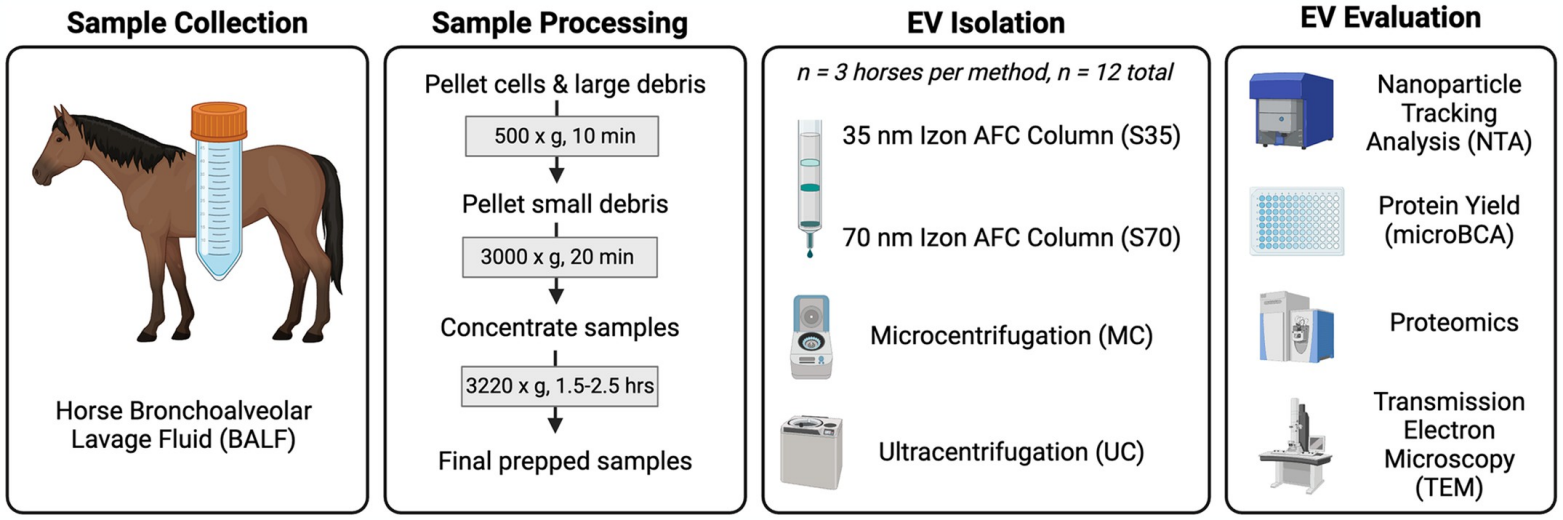
Overview of study design. Extracellular vesicles (EVs) were isolated from horse bronchoalveolar lavage fluid (BALF) (n = 3 horses) following sample processing via centrifugation (gray boxes in “Sample Processing” panel) using four different EV isolation methods, resulting in a total of 12 samples. EVs were evaluated and characterized through nanoparticle tracking analysis (NTA), micro bicinchoninic acid (microBCA) protein assay, proteomics, and transmission electron microscopy (TEM). Figure created with biorender.com.

An overview of the experimental design is presented in Fig 1.

### Sample collection and processing

#### Animal care and clinical respiratory score

This study was carried out in strict accordance with the recommendations in the Guide for the Care and Use of Laboratory Animals of the National Institutes of Health. All animal use procedures were approved by the North Carolina State University Institutional Animal Care and Use Committee (protocol #19–520 and #23–176). Horses included in this study were a convenience sample of university owned teaching animals that lived on pasture in the southeastern United States, received the same feed, and received no medications for at least 4 weeks prior to the study. Horses were identified as healthy or asthmatic based on history, physical exam, clinical respiratory score (S1 Table in S2 File) and bronchoalveolar lavage (BAL) cytology [27, 28]. BALF samples were obtained from one horse with severe equine asthma and two horses with mild/moderate equine asthma. None of these horses had clinical signs of asthma (cough, nasal discharge, nostril flare, abdominal effort, abnormal lung auscultation) at rest at the time of sample collection. Clinical scores are reported in S2 Table in S2 File.

#### BALF collection, processing, and cell differentials

BALF collection was performed as described previously [29]. Briefly, in order to relieve animal discomfort and anxiety, horses were placed under standing sedation with detomidine (0.005− 0.01 mg/kg IV) and butorphanol (0.02− 0.04 mg/kg IV), and 300 mL of warmed sterile saline was infused and re-aspirated in 150 mL aliquots, through a cuffed Bivona catheter. Average recovery volume for this procedure ranges from 180–220 mLs. Aliquots were pooled and a 10 mL sample was submitted for total cell count, cytopreparation, stained with a modified Wright’s solution, and a 400-cell differential count (cytospin) by a blinded veterinary clinical pathologists. Previously published healthy references ranges for equine BAL cytology are ≤ 5% neutrophils, ≤ 2% mast cells and ≤ 1% eosinophils. Horses with 6–19% neutrophils, > 2% mast cells and/or > 1% eosinophils are classified as having mild/moderate inflammation, and horses with >20–25% neutrophils are classified as having severe inflammation [28]. Cell differential data for this study are presented in S2 Table in S2 File.

#### BALF sample preparation for EV isolation

Cells were pelleted by centrifuging BALF at 500 x g for 10 minutes. The supernatant (cell-free BALF) was collected and stored at -20°C until all samples were collected. BALF samples were thawed, vortexed, and centrifuged at 3000 x g for 20 minutes at 4°C to pellet large debris. 20 mL of BALF from each horse was concentrated down to 2–8 mL using a 10 kDa MWCO centrifugal filter (Thermo Fisher Scientific 88528) (3220 x g for 1.5–2.5 hours at 4°C). Concentrated BALF was aliquoted in LoBind tubes (Eppendorf 022431081) and stored at -80°C until EV isolation.

### Extracellular vesicle isolation

#### 70nm and 35nm size exclusion chromatography

Concentrated BALF samples (1 mL) were thawed on ice and vortexed briefly. EVs were isolated using the Izon Automated Fraction Collector (AFC) with 35 or 70 nm Izon qEV1 Gen2 columns (Izon IC1-35; Izon IC1-70) per manufacturer’s protocol. The Izon AFC is a size-exclusion chromatography aid that collects buffer volumes and fractions based on user-input volumes. These volumes are measured by weight rather than by flow rate. The buffer volume was set at 3.3 mL and nine fractions of 700 μL each were collected to determine the EV elution profile across fractions (S1–S4 Figs in S1 File). 0.1 μm-filtered PBS (Thermo Fisher Scientific 14190144; VWR 89220–694) was used throughout the isolation process. EVs were stored in LoBind tubes (Eppendorf 022431081) at −80°C.

#### Microcentrifugation

Concentrated BALF samples (1–2 mL) were thawed on ice and vortexed briefly. To remove any small debris, the samples were centrifuged at 10,000 x g for 30 minutes at 4°C, and the supernatant was transferred to a new microcentrifuge tube. To pellet EVs, the supernatant was centrifuged at 21,000 x g for 60 minutes at 4°C. EVs were resuspended in 500 μL cold 0.1 μm-filtered PBS (Thermo Fisher Scientific 14190144; VWR 89220–694), aliquoted in LoBind tubes (Eppendorf 022431081), and stored at −80°C.

#### Ultracentrifugation

Concentrated BALF samples (1–2 ml) were centrifuged at 10,000 x g for 60 minutes to remove large vesicles. The supernatant was collected and centrifuged at 100,000 x g for 4 hours (36200 rpm, fixed angle rotor), and supernatant was removed by aspiration. EVs were re-suspended in 9 mL of PBS (Thermo Fisher Scientific 14190144) and centrifuged at 100,000 x g for 2 hours (36200 rpm, fixed angle rotor). EV pellets were resuspended in 50 μL of 0.02 μm-filtered PBS (Thermo Fisher Scientific 14190144; Millipore Sigma WHA68092102) and stored at -80°C.

### Extracellular vesicle characterization

#### Nanoparticle tracking analysis

Nanoparticle tracking analysis (NTA) was conducted to characterize potential variability in EV size and count distributions resulting from different isolation methods, while also informing size exclusion chromatography fractions selection, using previously published methodology [30–32]. EV samples from N = 3 horses per isolation method were evaluated using the Multiple—Laser ZetaView^®^ f-NTA Nanoparticle Tracking Analyzers System (Particle Metrix), including nine fractions per horse for each of the two size exclusion chromatography columns. Samples were diluted 1:10–1:10,000 (to ensure particle counts were in optimal range for measurement) in 0.02 μm filtered PBS (Thermo Fisher Scientific 14190144; Millipore Sigma WHA68092102), and 1 mL of each sample was injected for analysis. Measurements of EV size and count were performed in scatter scanning mode with sensitivity set to 80.

#### Total protein assay

Total protein in EV samples was quantified using a Pierce microBCA Protein Assay Kit (Thermo Fisher Scientific 23235) per manufacturer’s instructions. Samples were analyzed neat (no dilution, input volume of 50 μL in a half-area 96-well plate) to ensure samples fell within the standard curve of the assay. Absorbance at 562 nm was measured using a SpectraMax iD5 plate reader (Molecular Devices).

#### Sample preparation for proteomic profiling

To achieve sufficient protein concentrations for successful proteomic profiling, EV samples from the size exclusion chromatography and microcentrifugation methods were concentrated prior to proteomics analysis. For EV samples from the size exclusion chromatography method, fractions 3 and 4 (representing peak EV elution, S2–S4 Figs in S1 File) were pooled for each horse and concentrated from ~600–1000 μL to 100–200 μL using Amicon Ultra-2 centrifugal filters with 3 kDa MWCO (3220 x g for 30–60 minutes at room temperature) (Millipore Sigma UFC200324). Microcentrifugation samples were concentrated from ~400 μL to ~50 μL using Amicon Ultra-0.5 centrifugal filters with 3 kDa MWCO (14000 x g for 20 minutes at room temperature) (Millipore Sigma UFC500308). Concentrated samples were stored in LoBind tubes (Eppendorf 022431081) at -80°C until proteomic analysis.

#### Proteomic profiling

Samples (n = 3 per EV method) were dried down and resuspended in 8M urea, 50 mM ammonium bicarbonate (Sigma-Aldrich 09830). Next, they were reduced with 5mM Dithiothreitol (DTT; Fisher Scientific PIA39255) for 45min at 37ºC, alkylated with 15mM iodoacetamide (IAA; Fisher Scientific PIA39271) for 45min at room temperature. Samples were then diluted to > 1M urea, digested with trypsin (Promega V5113) overnight at 37ºC at an enzyme:protein ratio of 1:50. The resulting peptides were acidified to 0.5% trifluoracetic acid (TFA; Thermo Fisher Scientific 28904), and desalted using Pierce desalt spin columns (Thermo Scientific 89877). The samples were analyzed by LC/MS/MS using an Easy nLC 1200 coupled to a Qexactive HF mass spectrometer (Thermo Scientific). Samples were injected onto Aurora Ultimate TS column (75 μm id × 25 cm, 1.7 μm particle size; IonOpticks AUR3-15075C18) and separated over a 2 hr method. The gradient for separation consisted of 5–45% mobile phase B at a 250 nl/min flow rate, where mobile phase A was 0.1% formic acid in water (Fisher Scientific A117-50, Fisher W54) and mobile phase B consisted of 0.1% formic acid in 80% ACN (Fisher Scientific A955-4). The QExactive HF was operated in data-dependent mode where the 15 most intense precursors were selected for subsequent fragmentation. Resolution for the precursor scan (m/z 300–1600) was set to 120,000, while MS/MS scans resolution was set to 15,000. The normalized collision energy was set to 27% for HCD. Peptide match was set to preferred, and precursors with unknown charge or a charge state of 1 and ≥ 7 were excluded. Raw data were analyzed using Proteome Discoverer 3.1 and searched against the Uniprot Equus caballus (horse) database (UP000002281), which contained ~69,000 protein sequences, and appended to a common contaminants database (MaxQuant, 245 sequences). The proteomics datasets generated and analyzed in this study are available in the Proteomics Identification Database (PRIDE) repository under project identifier PXD054702.

#### Transmission electron microscopy

As recommended by the International Society for Extracellular Vesicles (ISEV), a representative sample isolated using the 35 nm size exclusion chromatography method was further visualized using electron microscopy. Visual characterization of EVs was performed using negative staining transmission electron microscopy (TEM) using previously published methods [30–32]. Glow-discharged formvar/carbon-coated 400 mesh copper grids (Ted Pella 01754-F) were suspended on sample droplets for ten minutes. Grids were then moved to deionized water for one minute, followed by 2% aqueous uranyl acetate stain for one minute. Grids were blotted and air dried before imaging. EVs were visualized using a JEM-1230 transmission electron microscope operating at 80 kV (JEOL USA inc.). Images were captured using a Gatan Orius SC1000 CCD camera equipped with Gatan Microscopy Suite Software (v. 3.10.1002.0).

#### Exo-check array

To validate purity of isolated EVs, an Exo-Check Array (System Biosciences EXORAY200B-4) was performed on a representative sample isolated using the 35 nm size exclusion chromatography method. The assay was carried out per manufacturer’s instructions using 160 μL of sample (~10 μg total protein) as input, developed using Avansta WesternBright Sirius HRP Substrate (Avansta K-12043-C20), and visualized using a BioRad ChemiDoc imager.

### Data analysis

#### Reproducibility

All analyses were performed in RStudio with R version 4.3.1 [33]. Packages used throughout the analysis included *tidyverse* [34], *janitor* [35], *openxlsx* [36], *patchwork* [37], *ggpubr* [38], and *rstatix* [39]. All input files, script, and output files are publicly available online via the Ragerlab Github [40].

#### Evaluating differences in total particle yield, particle size, and total protein yield between isolation methods

To adjust for differing concentration factors between samples and differing input and output volumes for each isolation method, particle and protein concentrations were normalized to the amount of starting raw BALF provided (20 mL). Prior to between-group statistical testing, NTA and microBCA assay data were tested for normality using qualitative (histograms, qqplots) and quantitative (Kruskal-Wallis test) approaches. A Friedman test was used to compare total particle yield, particle size characteristics, and total protein yield across isolation methods, with unique horse ID used as the factor for matching. Pairwise, paired Kruskal-Wallis tests were used for post-hoc testing to determine significant differences between pairs of methods within each endpoint.

#### Proteomic data processing

Prior to proteomic data analysis, data were processed to remove proteins with < 3 unique peptides and unannotated proteins. To account for variation in proteomic profiles across isolation methods, proteins were then filtered to include only those that were present in at least two out of three samples for at least one of the isolation methods. Resulting data were log2 transformed prior to downstream analyses.

#### Comparing EV-specific proteins, contaminating proteins, and cell surface marker proteins across isolation methods

Lists of EV-specific proteins and contaminating proteins were obtained from the MISEV2018 guidelines [41]. Data were queried for cell surface marker proteins that could potentially inform cell type of origin using string matches for gene symbols containing “CD” (cluster of differentiation), “LY6G” (lymphocyte antigen 6 family member G), “CCR” (C-C chemokine receptor), and “EPCAM” (epithelial cellular adhesion molecule) based on *a priori* hypotheses about potential cell origins of BALF EVs. Heatmaps of the number of samples in which a protein was detected or the average abundance of detected protein within each method were generated using the *pheatmap* package [42].

#### Determining significant differences in individual protein expression across isolation methods

Prior to between-group statistical testing, proteomics data (in log_2_) were tested for normality using qualitative (histograms, qqplots) and quantitative (Kruskal-Wallis test) approaches. A matched one-way ANOVA was used to compare relative expression of individual proteins between isolation methods, with pairwise, paired t-tests used for post-hoc testing.

#### Assessing overall differences in proteomic profiles across isolation methods

Two unsupervised machine learning methods were used to assess overall differences and similarities in proteomic profiles between samples and isolation methods: 1) hierarchical clustering based off Euclidean distances (*pheatmap* package [42]) and 2) principal component analysis (*factoextra* package [43]). Analyses were conducted on two datasets: (1) Proteins with no missing data (n = 62 proteins), and (2) Detection-filtered proteins, requiring each protein to be measured in at least two of the three samples in at least one of the methods (n = 564 proteins). Missing values were considered missing not at random—i.e., the protein was either not present in the sample or its abundance was below the limit of detection. For the detection-filtered dataset, missing values were imputed using GSimp [44] to allow for downstream analyses.

#### Characterizing enriched pathways across isolation methods

To understand enrichment of proteins associated with specific biological pathways, pathway analysis was conducted with Ingenuity Pathway Analysis (IPA) software. Prior to analysis, data were filtered within each isolation method to contain only proteins that were detected in two out of three samples for that method (S4 and S5 Tables in S2 File). Due to limited network mapping information specific to equine protein IDs, results were generated by mapping equine protein IDs to the human knowledgebase, which provided considerable overlap. For more detail surrounding coverage of horse proteins in the IPA human knowledgebase, see S6 Table in S2 File. For enrichment statistics, a modified Fischer’s Exact test was employed and corrected for multiple testing via the Benjamini Hochberg (BH) procedure, as previously described [30], and a BH p-value < 0.05 was considered significant. IPA results were exported for visualization in R.

## Results

### Total particle and protein yield differs significantly across isolation methods

**Fig 2:**
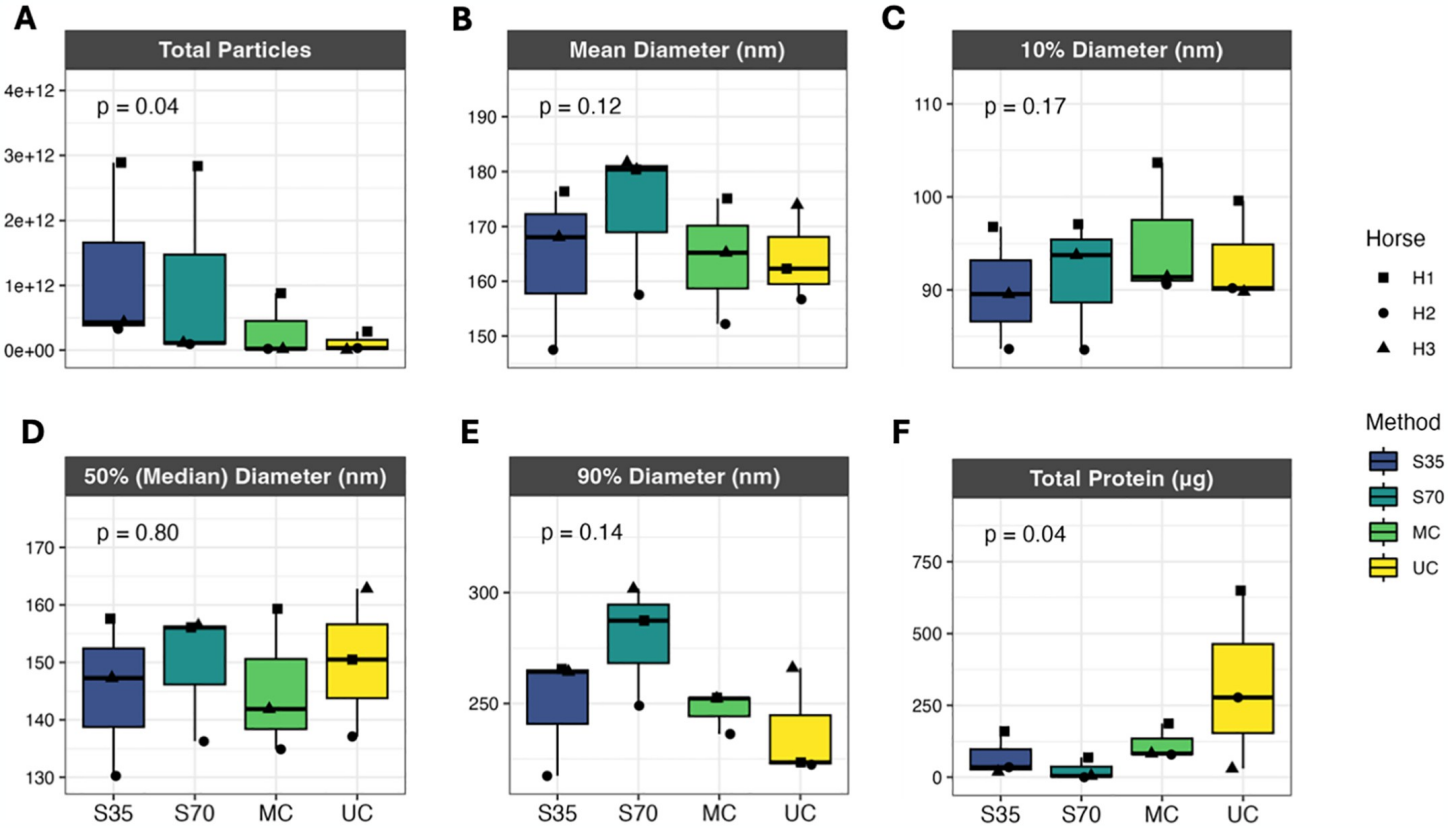
Particle characteristics of EV samples isolated from horse BALF using different isolation methods. Total particle yield, size (mean diameter, 10% diameter, 50% diameter, 90% diameter), and total protein yield (top left to bottom right panels) were compared across isolation methods using a Friedman test (matched within each unique horse) to obtain overall p-value followed by post-hoc pairwise, paired Wilcoxon tests. Only overall p-values are shown, as there were no significant pairwise comparisons between isolation methods. To adjust for differing concentration factors between samples and differing input and output volumes for each isolation method, total particles and total protein represent the yield from 20 mL unconcentrated BALF. S35 = size exclusion with Izon 35 nm column, S70 = size exclusion with Izon 70 nm column, MC = microcentrifuge, UC = ultracentrifuge. Size exclusion data represent the sum (total particles, total protein) or average (size) of fractions 3 and 4 (see supplement for fraction selection data).

To understand whether particle characteristics of EV samples were significantly different across different isolation methods, we compared total particle yield, particle size distribution, and total protein yield (Fig 2). Total particle and protein yield were significantly different across isolation methods by overall p-value (Fig 2A and 2F). In samples isolated using size exclusion chromatography, we observed a trend of higher total particle yield, in contrast with lower total protein. However, there were no significant differences between specific pairs of isolation methods within each endpoint (Fig 2). Size distributions were not significantly different across isolation methods (Fig 2B–2E). We also noted that variability for total particles was higher for the size-exclusion chromatography samples, while variability for total protein was higher for ultracentrifugation samples (Fig 2A). Mean total particle yield from 20 mL of unconcentrated horse BALF averaged from 1 x 10^11^ (ultracentrifugation) to 1.2 x 10^12^ (35 nm size exclusion column) (S3 Table in S2 File). Particles ranged from approximately 90 nm (10^th^ percentile) to 280 nm (90^th^ percentile) in diameter, with median diameter approximately 145–150 nm (S3 Table in S2 File), similar to EV size ranges reported in previous studies of mouse [18], human [19], and horse [45] BALF.

### Horse BALF EVs contain hundreds of unique proteins

A total of 1023 protein groups were identified (S4 Table in S2 File), representing 934 unique proteins, 44 of which were considered contaminants (representing proteins such as keratins from horses and humans and bovine proteins such as bovine serum albumin). After removing proteins with fewer than three unique peptides and unannotated proteins, 669 proteins remained in the dataset, representing 660 unique proteins (including 44 contaminants). Applying a filter that kept only proteins that were detected in two or more samples in at least one method reduced the number of proteins to 564 (556 unique) (S5 Table in S2 File). Only 62 proteins were detected in all three samples across all four isolation methods.

### Size exclusion chromatography yields purest EV samples

**Fig 3:**
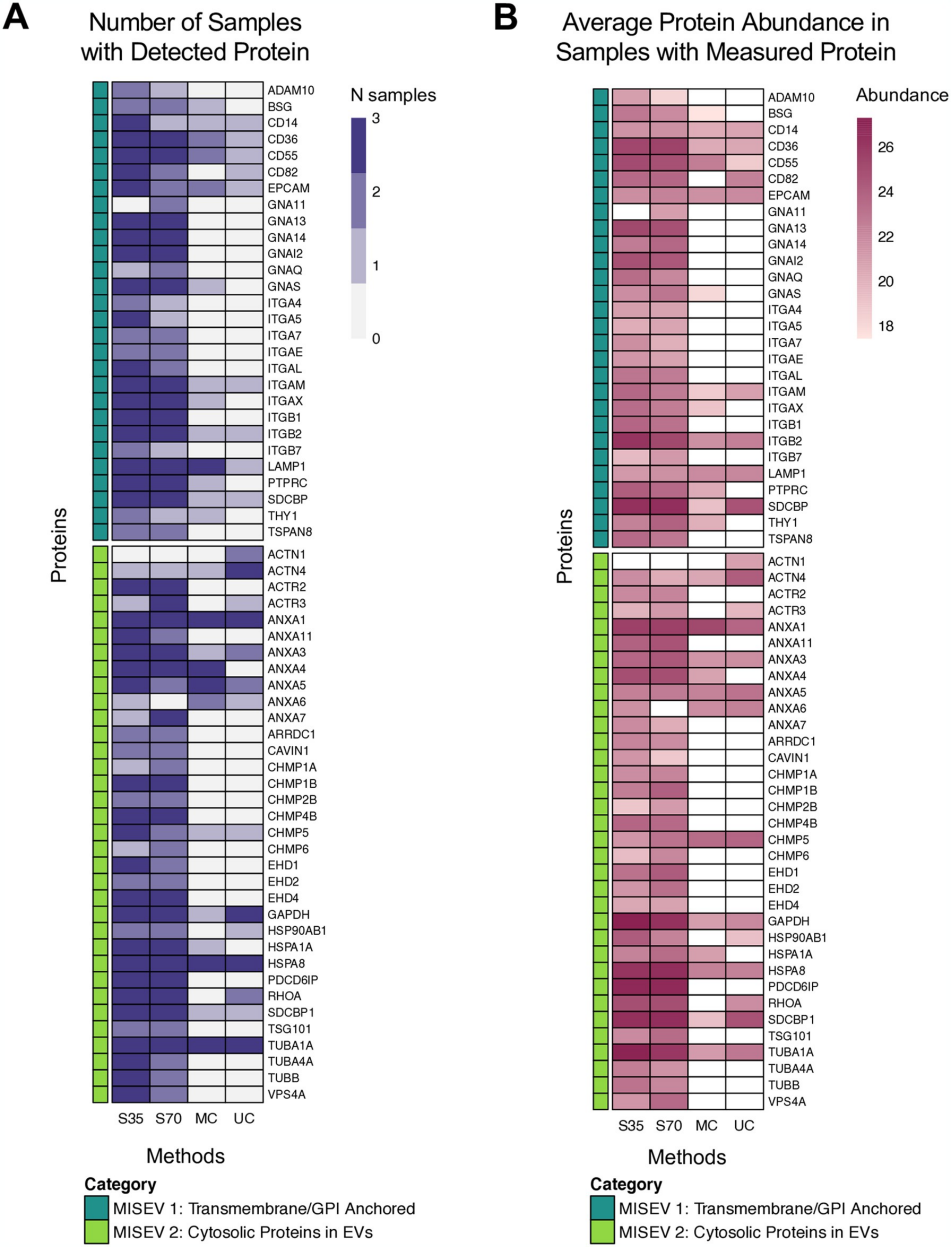
Presence and relative abundance of EV marker proteins in horse BALF EVs. (A) Number of samples with detectable levels of protein (out of a maximum of 3 per EV isolation method). (B) Average protein relative abundance per protein and EV isolation method for samples with detectable protein levels. Log2 relative abundance data were used as input for heatmap. White fill represents protein and method combinations with no detectable protein in any sample, resulting in an NA value for the mean. S35 = size exclusion with Izon 35 nm column, S70 = size exclusion with Izon 70 nm column, MC = microcentrifuge, UC = ultracentrifuge, MISEV = Minimal Information for Studies of Extracellular Vesicles.

**Fig 4:**
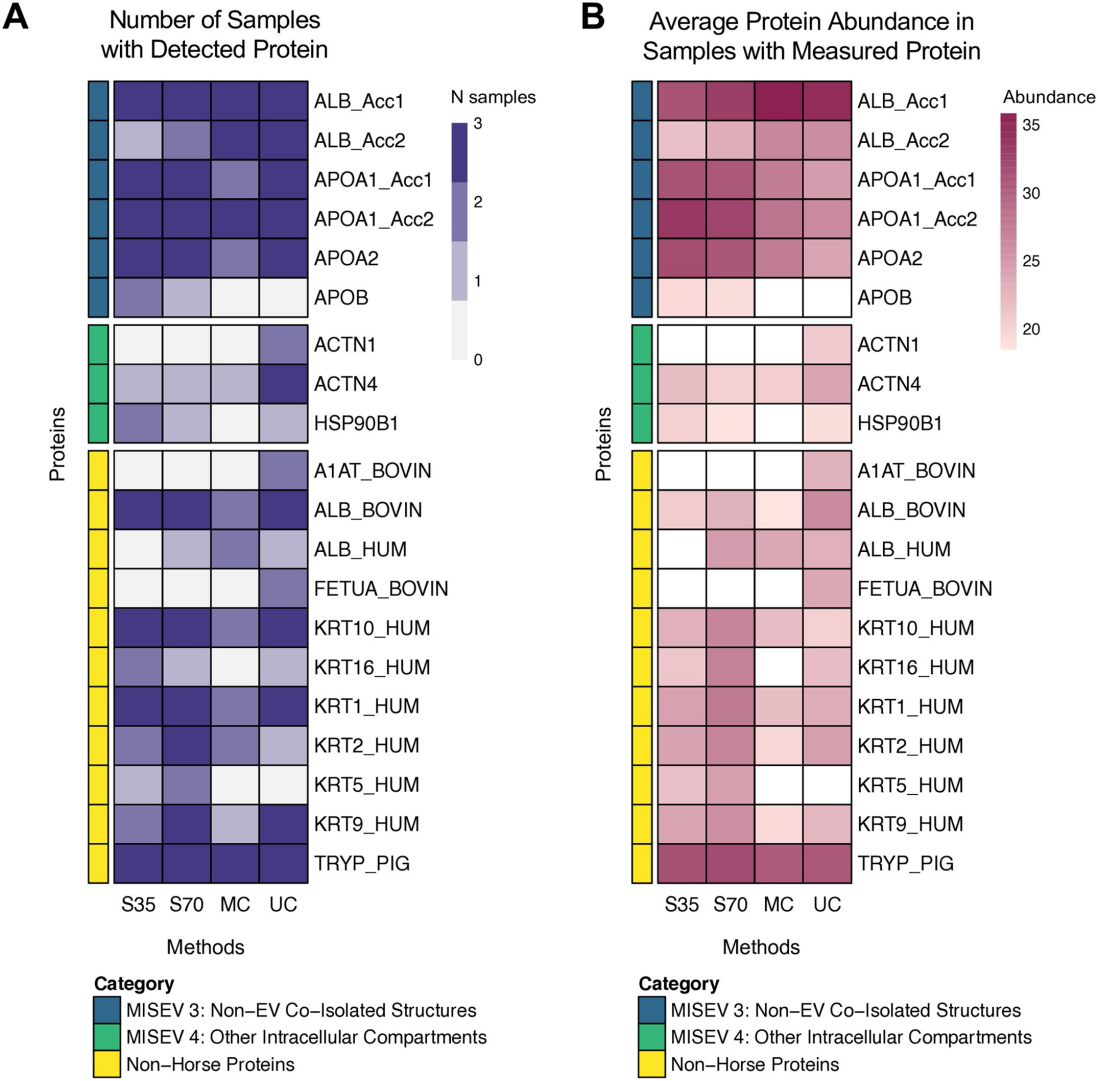
Presence and relative abundance of contaminant proteins in horse BALF EVs. (A) Number of samples with detectable levels of each contaminant protein (out of a maximum of 3 per EV isolation method). (B) Average relative abundance per protein and EV isolation method for samples with detectable protein levels. Log2 relative abundance data were used as input for heatmap. White fill represents protein and method combinations with no detectable protein in any sample, resulting in an NA value for the mean. Where there were two accessions mapping to the same protein,”_Acc1” and “_Acc2” were used to provide unique row names in cases of duplicate gene symbols. S35 = size exclusion with Izon 35 nm column, S70 = size exclusion with Izon 70 nm column, MC = microcentrifuge, UC = ultracentrifuge, MISEV = Minimal Information for Studies of Extracellular Vesicles.

We investigated the purity of EVs from each isolation method by referencing the 2018 MISEV guidelines [41], which provide lists of proteins considered positive markers for EVs (Category 1: transmembrane or GPI-anchored proteins; Category 2: cytosolic proteins found in EVs) and contaminants in EV preparations (Category 3: non-EV co-isolated structures; Category 4: proteins associated with other intracellular compartments). Detection and abundance of positive markers for EVs were notably higher in EVs isolated with both types of size-exclusion chromatography columns in comparison with EVs isolated using microcentrifugation or ultracentrifugation (Fig 3). Contaminants were detected in EV preparations across methods, with 4 out of 6 possible MISEV Category 3 and 3 out of 16 possible MISEV Category 4 contaminants detected (Fig 4A). The highest abundance contaminants were horse albumin (particularly in centrifugation samples) and apolipoproteins (particularly in size exclusion chromatography samples) (Fig 4B). To validate EV isolation, we performed transmission electron microscopy on a sample isolated with the 35 nm size exclusion column (S5 Fig in S1 File), which demonstrated clear presence of vesicles and that vesicles were in a similar size range to the diameters measured using NTA. We also performed an Exo-Check Array to assess presence of common EV-associated markers (S6 Fig in S1 File). Signal was strongest for ANXA5, TSG101, ICAM, and ALIX, with weaker signal from CD63, EPCAM, CD81, and GM130 (a cellular contamination marker).

### EVs isolated with size exclusion chromatography retain cell surface markers potentially informing cell type of origin

**Fig 5:**
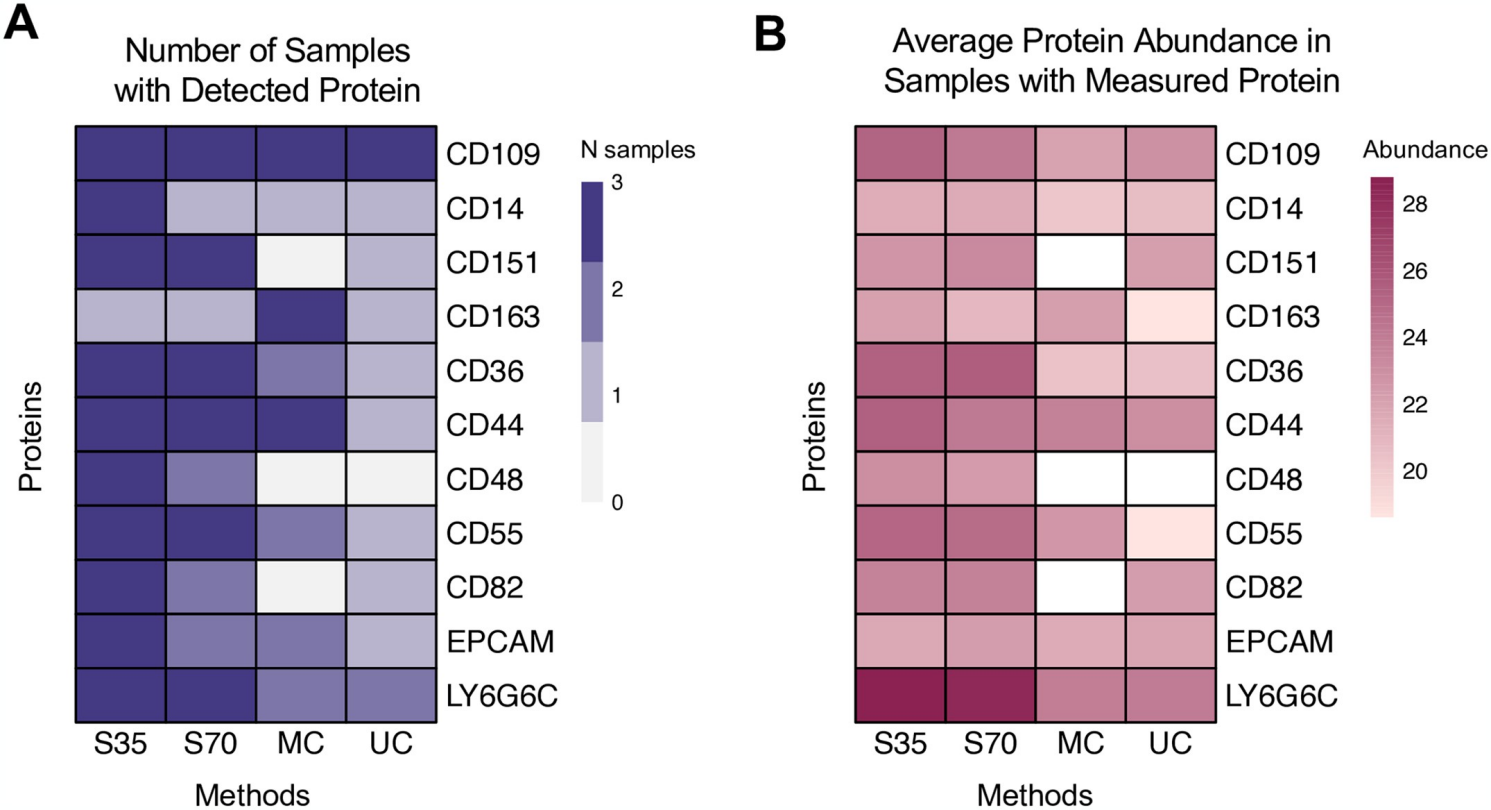
Presence and relative abundance of cell surface marker proteins potentially informing cell type of origin in horse BALF EVs. (A) Number of samples with detectable levels of protein (out of a maximum of 3 per EV isolation method). (B) Average protein relative abundance per protein and EV isolation method for samples with detectable protein levels. Log2 relative abundance data were used as input for heatmap. White fill represents protein and method combinations with no detectable protein in any sample, resulting in an NA value for the mean. S35 = size exclusion with Izon 35 nm column, S70 = size exclusion with Izon 70 nm column, MC = microcentrifuge, UC = ultracentrifuge.

EVs are thought to express surface markers that reflect their cell-of-origin and can enable investigation of cell type-specific EV signatures; however, potential candidates for these markers, particularly in the respiratory tract, have not been quantified directly through EV proteomics, as they are difficult to detect. Therefore, we next assessed detection and abundance of potential candidate cell surface markers across isolation methods (Table 1). These potential cell surface markers included 9 CD’s, EPCAM, and LY6G6C, with references supporting their utility in informing cells of origin provided in Table 1. We found that surface marker detection and abundance was higher in EVs isolated with size exclusion chromatography (Fig 5). LY6G6C, expressed primarily by neutrophils and other granulocytes [46, 47], had the highest abundance out of all the surface markers, suggesting granulocytes as a dominant source of EVs within horse BALF.

**Table 1:** Cell types associated with expression of cell surface markers detected in horse BALF EVs.

Protein	Cell Types Associated with Expression	Reference(s)
CD109	A subset of hematopoietic stem and progenitor cells; activated platelets and T cells; endothelial cells	[48, 49]
CD14	Primarily macrophages; to a small extent neutrophils and dendritic cells; can also be expressed in other cell types but likely at much lower levels	[50–52]
CD151	T cells, endothelial cells, epithelial cells	[53]
CD163	Monocytes and macrophages	[54]
CD36	Many cells and tissues, including macrophages and respiratory epithelial cells	[55]
CD44	Primarily lymphohematopoietic cells, but can also be expressed on respiratory epithelial cells	[56]
CD48	Primarily hematopoietic cells	[57]
CD55	Primarily epithelial cells, stroma, and endothelial cells; can also be expressed on lymphoid cells	[58, 59]
CD82	Primarily leukocytes and platelets; one study found detection on smooth muscle cells, but limited literature	[60]
EPCAM	Primarily epithelial cells	[61]
LY6G6	Primarily granulocytes (specifically, neutrophils)	[62]

### Protein profiles differ significantly across isolation methods

Of the 62 proteins detected in all three samples across all four isolation methods, 48 showed significantly different abundances across isolation methods by overall p-value (S7 Table in S2 File). There were significant differences in abundance by pairwise comparisons between methods for 20 proteins (S7 Fig in S1 File), with proteins such as apolipoprotein 1 (APOA1), ceruloplasmin (CP), immunoglobulin heavy constant mu (IGHM), J chain (JCHAIN), and antithrombin-III (SERPINC1) more abundant in EVs isolated with centrifugation in comparison with size exclusion chromatography. In contrast, proteins including clusterin (CLU), surfactant protein B (SFTPB), and tubulin alpha-1A chain (TUBA1) were less abundant in EVs isolated with centrifugation in comparison with size exclusion chromatography (S7 Fig in S1 File).

**Fig 6:**
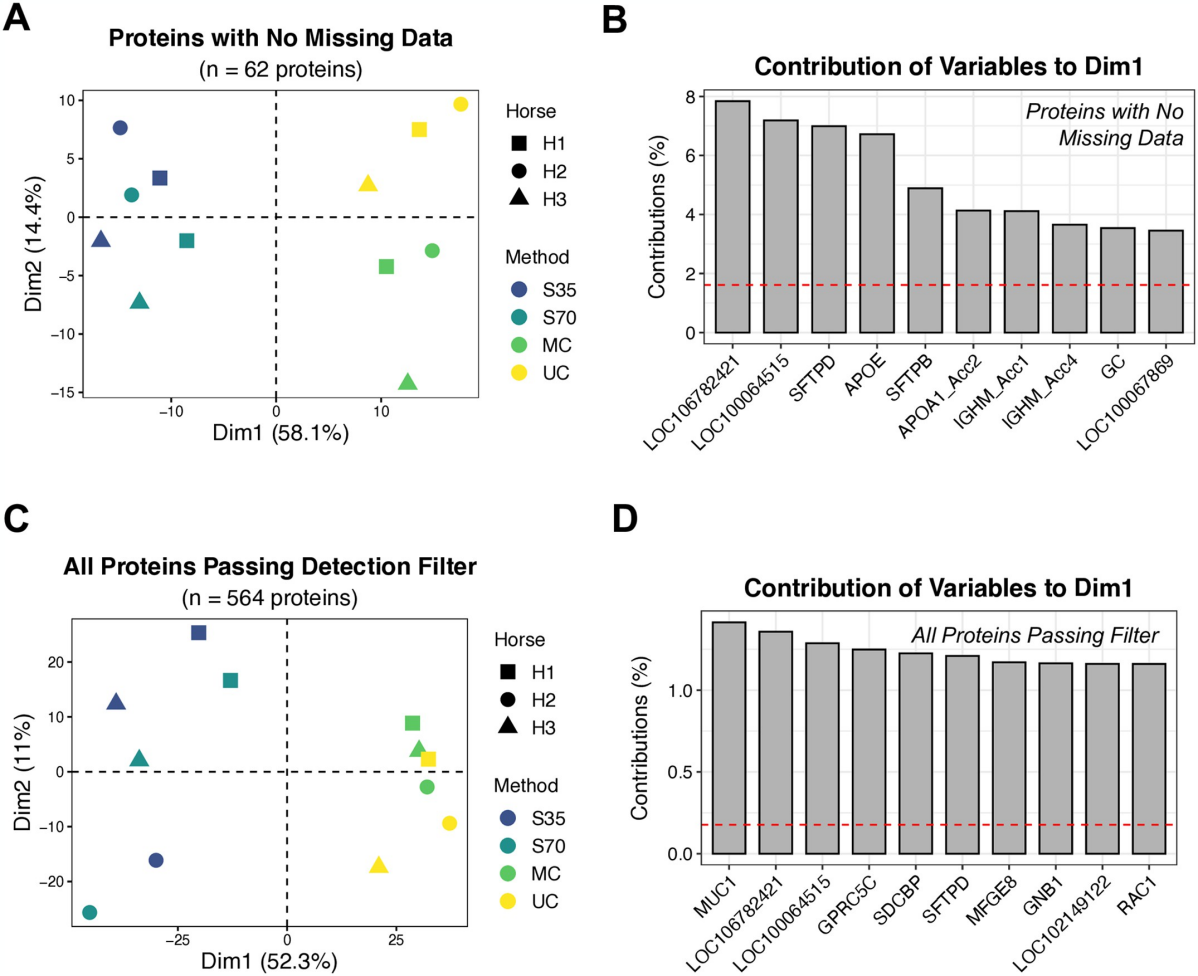
Principal component analysis of BALF EV protein to understand similarities between isolation methods. (A) PCA plot generated using proteins with no missing data (n = 62 features). (B) Contributions of the top 10 highest contributing proteins to the first PCA dimension (Dim1) in figure A. (C) PCA plot generated using protein data for proteins passing the detection filter (detected in at least 2 horses for at least one isolation method). Missing data were imputed prior to running PCA (n = 564 features, including the n = 62 features in figures A and B). (D) Contributions of the top 10 highest contributing proteins to the first PCA dimension (Dim1) in figure C. Log2 abundance data were used as input for PCA analyses. S35 = size exclusion with Izon 35 nm column, S70 = size exclusion with Izon 70 nm column, MC = microcentrifuge, UC = ultracentrifuge.

Examining overall protein signatures through unsupervised machine learning, we found that samples clustered largely by isolation method, with EVs isolated using size exclusion chromatography clustering separately from EVs isolated using centrifugation methods (Fig 6A and 6C and S8 and S9 Figs in S1 File) in both PCA and hierarchical clustering analyses. PCA on the dataset containing proteins with no missing data (Fig 6A) demonstrated clear separation between the two centrifugation methods but did not show clear separation within the size exclusion chromatography samples. Conversely, PCA on the dataset with all proteins passing a more relaxed detection filter (and thus had some missingness that required data imputation) (Fig 6C) did not show separation between centrifugation methods, yet it did show separation by horse within the size exclusion chromatography samples. Hierarchical clustering results also demonstrated clustering first by individual horse, then by column size with EVs isolated by size exclusion chromatography method, suggesting that inter-horse variability is greater than variability between the two column sizes (S8 and S9 Figs in S1 File).

To further understand proteins driving the separate clustering of size exclusion chromatography and centrifugation samples, we next examined proteins contributing to variation in the first dimension of the PCA (Fig 6B and 6D and S10 Fig in S1 File). For the dataset containing proteins with no missing data, expression levels of proteins such as surfactant proteins B and D (SFTPB, SFTPD) and apolipoproteins A1 and E (APOA1, APOE) were included in the list of the top 10 proteins contributing to variance explaining separation between the size exclusion and centrifugation methods (Fig 6B). For the detection-filtered dataset, SFTPD was still in the top 10 proteins contributing to separation between the size exclusion and centrifugation methods, alongside proteins such as mucin 1, G protein-coupled receptor class C group 5 member C (GPRC5C), syndecan binding protein (SDCBP), and others (Fig 6D).

### Top-ranking canonical pathways are largely shared across isolation methods

**Fig 7:**
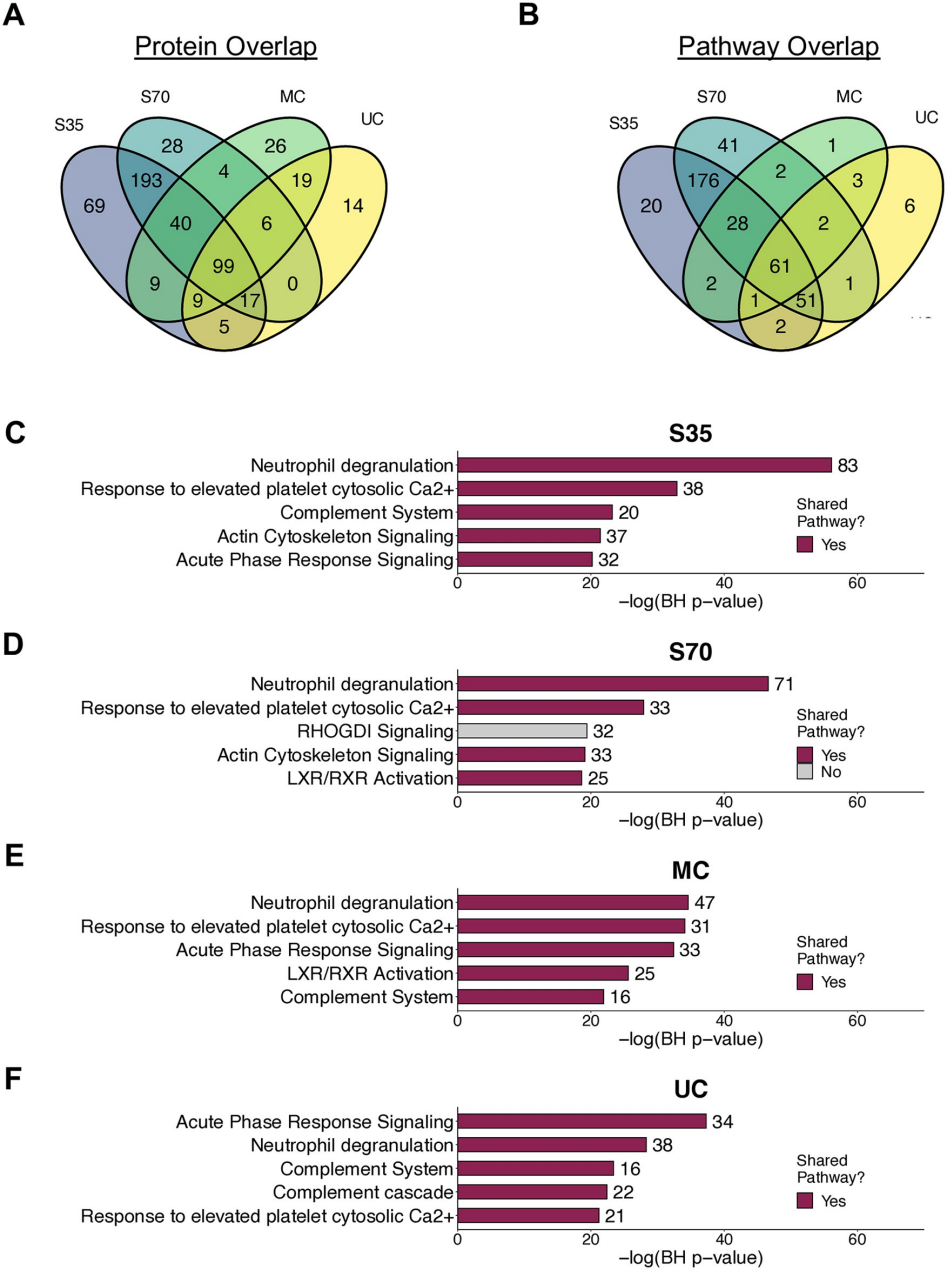
Overlap in protein expression and enriched pathways across EV isolation methods. (A) Overlap in proteins detected across methods, where a protein was considered detected if it was measured in 2 out of the 3 samples for that isolation method. (B) Overlap in significantly (BH p < 0.05) enriched canonical pathways across isolation methods. (C-F) Top 5 pathways by BH p-value for each isolation method, with number of proteins associated with that pathway annotated next to each bar. Magenta bars indicate that this pathway was significantly enriched (BH p < 0.05) in EVs from all isolation methods. S35 = size exclusion with Izon 35 nm column, S70 = size exclusion with Izon 70 nm column, MC = microcentrifuge, UC = ultracentrifuge.

To gain deeper insight into potential biological processes impacted by proteins contained in EVs isolated across each method, pathway enrichment analysis was carried out using proteins detected in 2 out of 3 samples (“detected”) for each isolation method. EVs isolated using the 35 nm size exclusion column contained the highest number of detected proteins (n = 441), followed by the 70 nm size exclusion column (n = 357), microcentrifugation (n = 212), and ultracentrifugation (n = 169) (S6 Table in S2 File). A total of 99 proteins were detected across all isolation methods, with the two size exclusion chromatography column sizes sharing a large number of detected proteins (n = 193) (Fig 7A). Similar patterns were observed when considering significantly enriched canonical pathways generated using these protein lists as input (Fig 7B). For example, 61 significantly enriched pathways were shared across all isolation methods, while 176 were shared only between the two size exclusion chromatography methods. Many of the most significantly enriched pathways were shared across all methods (Fig 7C–7F). For example, neutrophil degranulation was the most significantly enriched pathway in samples isolated using size exclusion chromatography and ultracentrifugation and the second most significantly enriched pathway in samples isolated using microcentrifugation (Fig 7C–7F). Other shared enriched pathways included response to elevated platelet cytosolic Ca^2+^, acute phase response signaling, and pathways related to coagulation and the complement system (Fig 7C–7F).

## Discussion

This study was designed to address the need for transparent methodological comparisons of EV isolation techniques, using equine BALF samples as a model biospecimen that can inform respiratory biology and toxicological responses in humans. Use of equine samples with high sample and cell yield volumes enabled extensive comparison across four isolation methods—size exclusion chromatography with two pore sizes (35 and 70 nm), microcentrifugation (21,000 x g), and ultracentrifugation (100,000 x g). Here, we reported, in detail, the sample and volume requirements for each EV isolation step and downstream assays, and we described resulting EV characteristics including proteomic signatures, which were significantly different across isolation approaches (summarized in Table 2). The following represent primary findings from this study: First, the physical characteristics of EV particles differed according to the isolation method. Second, size exclusion chromatography techniques yielded relatively higher sample purity and yield, while simultaneously capturing the greatest landscape of protein diversity, suggesting the highest EV isolation specificity. Third, measured EV protein cargo differed according to isolation method, including cell surface markers informing cell-of-origin. Lastly, EV protein signatures were enriched for processes involved in cell signaling and immune cell communication, suggesting an important role for EVs in respiratory signaling and intercellular communication.

**Table 2:** Summary of experimental timing and horse BALF EV characteristics across four different EV isolation methods. This table includes information on EV isolation method characteristics across two different size exclusion chromatography approaches and two different centrifugation methods, as delineated by the columns. Pluses indicate the relative amount of each characteristic, with more pluses (++++) indicating that method had a higher value for that characteristic than other methods (+). Rows describe the following: (A) the time required for each EV isolation method (note that for SEC, EV isolation time is approximately 1 hour; however, cleaning the column for reuse for the next sample takes approximately 1 additional hour); (B) the resulting particle and (C) protein yield (reflective of yield from 20 mL unconcentrated horse BALF, where 0 indicates below the limit of detection for microBCA assay); (D) EV quality, as assessed through particle yields relative to protein yields and presence of EV and contaminant markers; and (E) number of proteins detected in at least 2 out of 3 samples within each isolation method.

	Size Exclusion Chromatography		Centrifugation	
	35 nm SEC	70 nm SEC	Microcentrifugation	Ultracentrifugation
**A. Time**	++ 1–2 hours	++ 1–2 hours	++ 2 hours	++++ 7 hours
**B. Particle Yield**	++++ 3.3E11–2.9E12	+++ 9.3E10–2.8E12	++ 1.7E10–8.8E11	+ 6.0E9–2.9E11
**C. Protein Yield (μg)**	++ 19–160	+ 0–69	+++ 79–187	++++ 30–649
**D. EV Quality**	++++	++++	++	++
**E. Number of Proteins Detected**	++++ n = 441	+++ n = 357	++ n = 212	+ n = 169

Particle characteristics that differed significantly across isolation methods were total particle count and total protein yield, with the 35 nm size exclusion chromatography column yielding the most particles, while ultracentrifugation yielded the most total protein. Our finding that increased total protein yield was not indicative of increased total particle count has implications for reporting of EV input into specific assays, as EV input for downstream assays is often described in units of total protein as a proxy for EV count. This approach could lead to reproducibility issues, as different isolation methods resulted in differing total protein, which was not reflective of total EVs or EV cargo. We hypothesized that the higher total protein yield in ultracentrifugation samples may be due to higher levels of contaminating proteins, such as horse albumin. In support of this hypothesis are our findings that although contaminants were detected in all isolation methods, centrifugation methods resulted in higher albumin abundance and lower protein diversity (Table 2). However, samples isolated with size-exclusion chromatography had higher relative expression of apolipoproteins, demonstrating that no method produces completely contaminant-free isolates. When discussing contaminants, it is also important to acknowledge the growing body of literature describing the EV corona (the molecules surrounding the EV surface) and its importance for EV function [63–66]. Proteins that have traditionally been considered contaminants, such as albumin and apolipoproteins, are now recognized as also part of the corona, complicating assessment of EV purity using these markers. Therefore, because size exclusion chromatography was able to capture the greatest diversity of proteins (in terms of number of unique proteins measured) while at the same time, had the lowest total protein, we found that size exclusion chromatography represented the method yielding the highest sample purity and sensitivity in proteomic measurements for EV isolates from BALF.

Interestingly, we generally did not detect the presence of tetraspanins CD9, CD63, or CD81 in our proteomics dataset, though we did observe some signal for CD63 and CD81 using an Exo-Check Array. This could be because small, transmembrane proteins can be difficult to identify in proteomics and may not pass unique peptide detection filters (for example, we detected CD81, but only 2 unique peptides were identified), these markers are less abundant in BALF EVs than EV populations from other biological matrices or in comparison to the abundance of other proteins in BALF, or due to species mismatch in the Exo-Check array (human vs horse). Because these proteins have historically been considered as prototypical markers of EVs [67], they have been used both to validate isolations and as capture antibodies in EV immunoaffinity assays, such as bead-based pull down or flow cytometry. However, recent studies suggested that, even within EVs from a single sample, surface marker expression is heterogenous and that CD9, CD63, and CD81 may not be appropriate pan-EV markers [19, 68–71]. Studies have also demonstrated that these markers are not essential for EV uptake, cargo composition, or cargo delivery [72, 73]. Our results support the idea that expression of these specific tetraspanins is highly dependent on the source of the EVs and further demonstrate that use of assays relying on these markers may not be suitable for respiratory EVs. However, we successfully detected many other transmembrane proteins, including those considered general markers of EV presence by MISEV, such as tetraspanin 8 (TSPAN8) and integrin family proteins, alongside transmembrane proteins associated with specific cell types. Transmembrane proteins were detected at a greater frequency and abundance in samples isolated using size exclusion chromatography, in agreement with previous studies demonstrating that the shear forces generated during centrifugation isolation can damage EVs and reduce their functionality [74, 75]. Using isolation methods that maintain the integrity of the EV membrane and transmembrane proteins is particularly important in identifying EV cell-of-origin, as investigation of cell-type specific EV signatures is typically conducted through cell surface marker characterization and immunocapture. Characterizing cell-type specific EVs is an active area of research, though no studies to our knowledge have experimentally identified candidate EV cell surface markers for EV cell-of-origin studies in the respiratory tract. Here, we identified a number of these markers, including CD14 (macrophages/monocytes), CD163 (macrophages), EPCAM (epithelial cells), and LY6G6C (neutrophils), which inform cells likely emitting EVs in the respiratory tract. However, flow cytometric analyses are needed to validate the specificity of these markers, their presence at the EV surface, and the percentage of vesicles expressing each marker.

In addition to differences in cell surface marker detection and expression, we also found that EV proteomic signatures were significantly different across the four different isolation methods. Therefore, it is important to consider isolation methods when comparing expression of specific EV proteins or EV proteomic signatures between studies. Despite these differences, several of the most enriched canonical pathways were shared across methods, suggesting that some of the EV proteomic signature is retained regardless of isolation method. Most notably, these pathways were related to neutrophil degranulation, acute response signaling, and the complement system, demonstrating a role for EVs in innate immune signaling pathways in the respiratory tract. These findings were also in agreement with the detected cell-type specific surface markers, which were associated with neutrophils, macrophages, and other hematopoietic cells. When considering applicability of these findings to humans and model organisms such as rodents, it is also important to acknowledge species differences in respiratory immune cell populations. In human and rodent BALF, macrophages are the predominant cell type, while in horse BALF, both macrophages and lymphocytes represent large proportions of the cellular population. Therefore, comparison across organisms is needed to more fully assess agreement between species, and cell-type specific findings should be interpreted with caution when considering applicability of our findings to BALF from other species.

An important limitation of this study is that we assessed one type of bulk EV cargo in one respiratory sample type in one animal model. Optimizing EV isolation and comprehensively characterizing the EV cargo landscape in bronchoalveolar lavage fluid and other respiratory tract samples across varying models, such as human nasal lavage fluid and induced sputum, both in bulk and by cell-of-origin, represents a critical knowledge gap in understanding respiratory EV biology. This will also allow for direct comparison between human and horse respiratory EV biology, aiding in assessment of horses as a model organism for human respiratory disease and toxicant exposure. We also recognize that this study was performed with relatively a relatively low sample size, which may explain why main effect differences were detected in particle number and protein concentration, but no pairwise differences were identified. Future studies with larger sample sizes will enable the investigation of interindividual variation in EV cargo as well as the association between EV cargo and covariates such as age, sex, disease state, and environmental exposure. Additionally, limited sample material prevented us from imaging all samples with TEM and from performing Exo-Check Arrays on all samples. Future studies using larger input volumes will enable more thorough evaluation across a larger number of EV validation assays.

Taken together, this study demonstrates successful in-depth proteomic profiling of respiratory EVs, which enabled us to identify size exclusion chromatography as the isolation method generating the purest EV isolates from bronchoalveolar lavage fluid. Our results highlight the importance of EV isolation method in assessment of downstream EV cargo and provide essential baseline EV proteomic profiles needed to characterize potential EV functions and cell surface markers for cell-of-origin studies in the respiratory tract. This study also uniquely harnesses benefits of animal model choice, with the large volume of BALF collection in horses permitting a detailed, systematic assessment of EV cargo across isolation methods, which would be more difficult in other model organisms. Overall, this study paves the way for future investigations of the role of EVs in respiratory immune biology, disease pathogenesis, and response to environmental exposures and highlights the horse as an optimal model for study of respiratory EVs.

## Supporting information

S1 File Supplementary figures supporting fraction selection for EVs isolated with size-exclusion chromatography, EV validation via transmission electron microscopy, and differential protein expression between EVs isolation with different methods. (DOCX)

S2 File Supplementary tables describing equine health, summary statistics for particle characteristics, raw and imputed proteomics data, and full statistical results. (XLSX)
