# Supplementary material for: Respiratory extracellular vesicle isolation optimization through proteomic profiling of equine samples and identification of candidates for cell-of-origin studies
Source: PLoS One. 2025 Jan 24;20(1):e0315743. doi: 10.1371/journal.pone.0315743 (PMC11760557; doi:10.1371/journal.pone.0315743)
Supplement: S1 File — (DOCX) [file pone.0315743.s001.docx]

**Supplemental Figures** for: “Respiratory extracellular vesicle isolation optimization through proteomic profiling of equine samples and identification of candidates for cell-of-origin studies”

|  |
| --- |
| **Figure S1. Size exclusion chromatography fraction collection volumes.** Table describing the volume from the start of collection each fraction represents. Volumes are applicable to samples isolated with both the 35 and 70 nm qEV columns. |

|  |
| --- |
| **Figure S2. Fraction selection for 35 nm qEV1 column**. Total particle yields from each fraction are displayed for all three horses (A, mean ± standard deviation) or each individual horse (B). Fractions were collected with 3.3 mL buffer volume and 700 µL per fraction. For more detail on fraction volumes, see Figure S1. |

|  |
| --- |
| **Figure S3. Fraction selection for 70 nm qEV1 column**. Total particle yields from each fraction are displayed for all three horses (A, mean ± standard deviation) or each individual horse (B). Fractions were collected with 3.3 mL buffer volume and 700 µL per fraction. For more detail on fraction volumes, see Figure S1. |

| 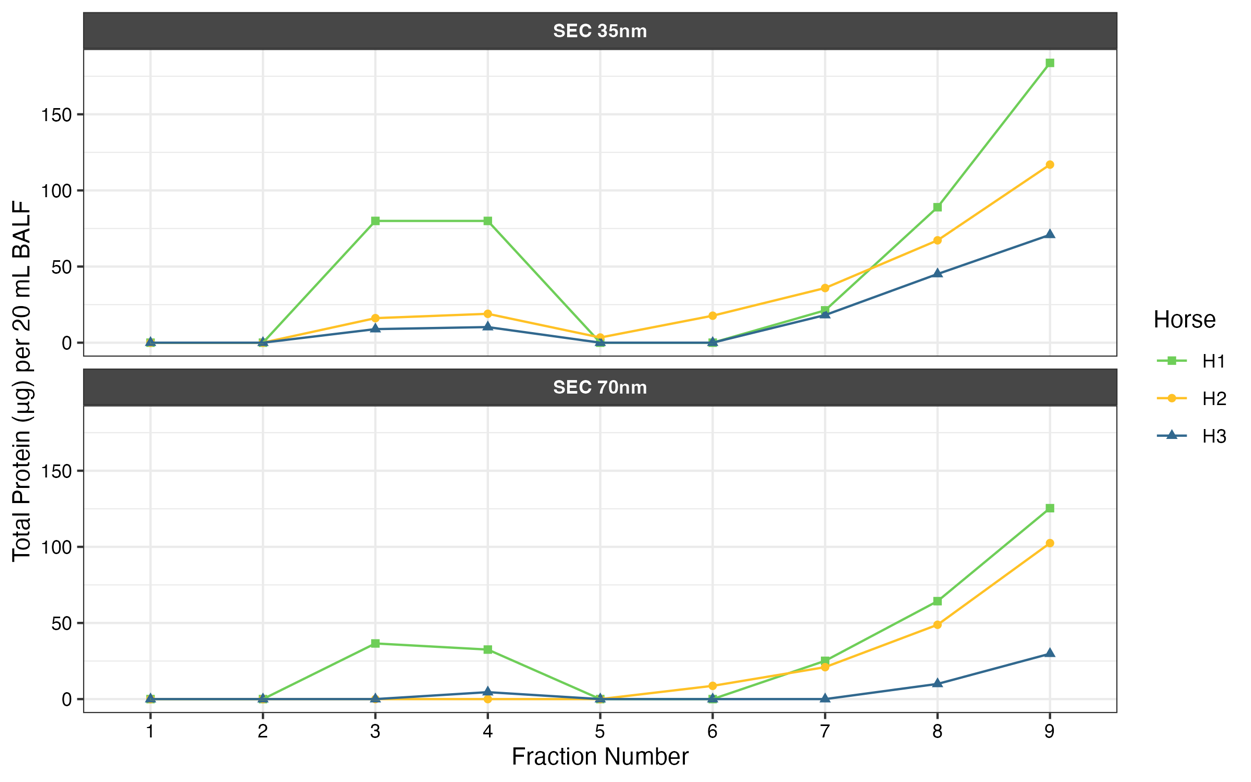 |
| --- |
| **Figure S4. Total protein per fraction.** Total protein from each fraction for the 35 nm or 70 nm size-exclusion column. Fractions were collected with 3.3 mL buffer volume and 700 µL per fraction. For more detail on fraction volumes, see Figure S1. |

|  |
| --- |
| **Figure S5. Transmission electron microscopy images of extracellular vesicles.** EVs samples representing fractions 3 (F3) and 4 (F4) from one horse (H1) were visualized using transmission electron microscopy at a range of magnifications. |

|  |
| --- |
| **Figure S6. Human Exo-Check Array for horse extracellular vesicle markers.** (A) Table summarizing sequence alignment as determined using the Ensembl Genome Browser to compare between horse and human sequences and whether or not each protein was detected in the processed proteomics dataset in the manuscript. (B) Exo-Check Array results for proteins considered EV markers, contaminant markers, and controls for assay performance. |

| 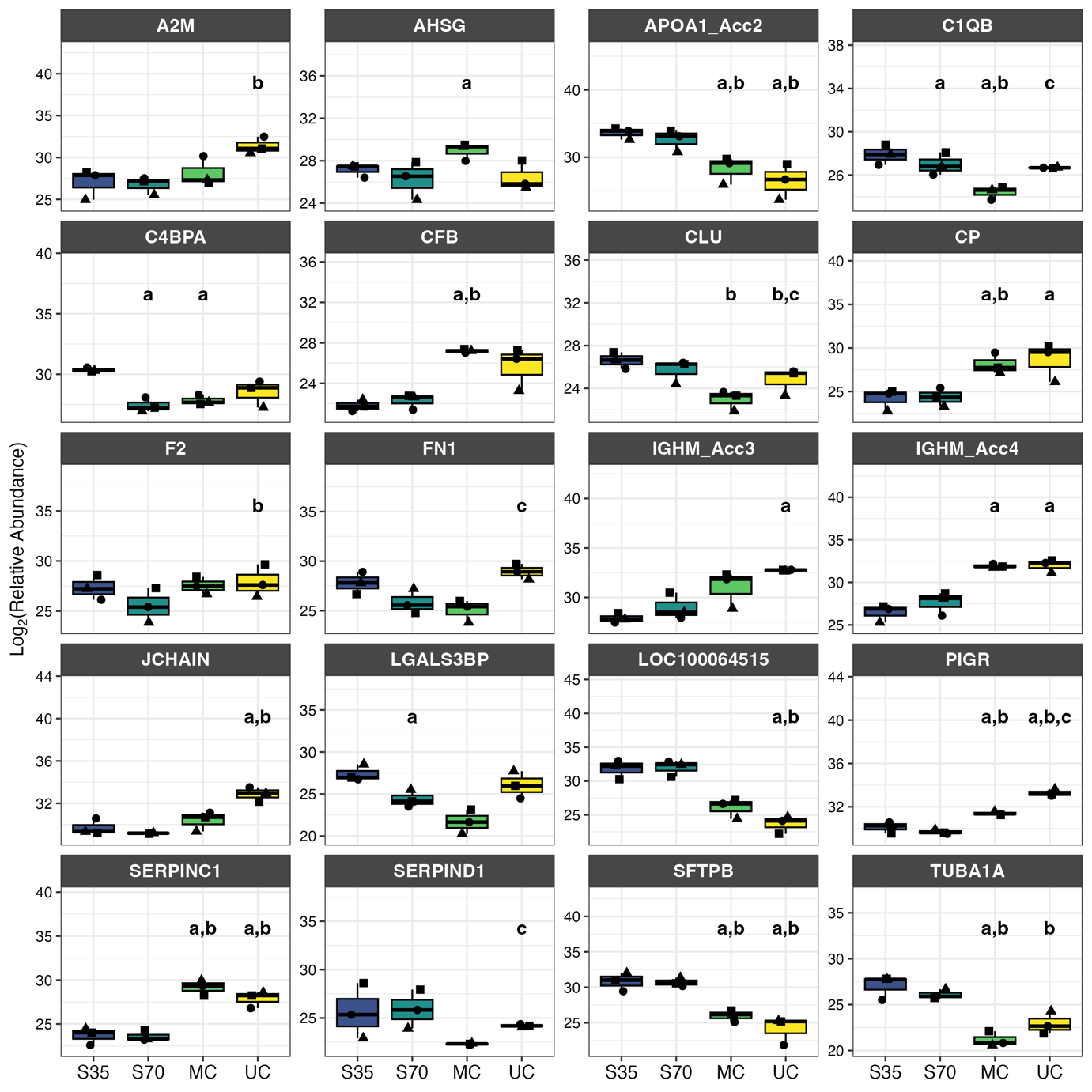 |
| --- |
| **Figure S7**. **Proteins with significantly different expression across isolation methods.** Overall significance was determined using a matched one-way ANOVA with pairwise, paired t-tests used for post-hoc testing. Benjamini-Hochberg correction was applied to account for multiple hypothesis testing. Letters indicate significance of at least p < 0.05 in comparison with (a) 35 nm size exclusion column, (b) 70 nm size exclusion column, and (c) microcentrifugation (c). For example, the “b” annotation over the UC column in the first graph (A2M) indications that expression was significantly different between 70 nm size exclusion column and ultracentrifugation. S35 = size exclusion with Izon 35 nm column, S70 = size exclusion with Izon 70 nm column, MC = microcentrifuge, UC = ultracentrifuge. |

|  |
| --- |
| **Figure S8. Hierarchical clustering of samples for proteins detected in all samples.** Log_2_ data were used as input with no scaling applied. S35 = size exclusion with Izon 35 nm column, S70 = size exclusion with Izon 70 nm column, MC = microcentrifuge, UC = ultracentrifuge. |

|  |
| --- |
| **Figure S9. Hierarchical clustering of samples for proteins detected at least two samples in at least one method.** Imputed, log_2_ data were used as input with no scaling applied. S35 = size exclusion with Izon 35 nm column, S70 = size exclusion with Izon 70 nm column, MC = microcentrifuge, UC = ultracentrifuge. |

|  |
| --- |
| **Figure S10.** **Principal component analysis variable contribution.** Variable contributions for the top seven variables generated by running principal component analysis on (A) only proteins with no missing data (n = 62) or (B) all data passing the detection filter (2 detections in at least one method) (n = 564). Missing values were imputed prior to PCA. |
